# The bacterial biocontrol agent *Paenibacillus alvei* K165 confers inherited resistance to *Verticillium dahliae*

**DOI:** 10.1093/jxb/erab154

**Published:** 2021-04-08

**Authors:** Danai Gkizi, Anna González Gil, Alonso J Pardal, Sophie J M Piquerez, Chrysi Sergaki, Vardis Ntoukakis, Sotirios E Tjamos

**Affiliations:** 1 Laboratory of Plant Pathology, Agricultural University of Athens, Athens, Greece; 2 School of Life Sciences, University of Warwick, Coventry, UK; 3 Warwick Integrative Synthetic Biology Centre, University of Warwick, Coventry, UK; 4 Queen’s University, Canada

**Keywords:** Arabidopsis, biocontrol agents, fungal pathogens, resistance, symbiosis, *Verticillium*

## Abstract

The biocontrol agent *Paenibacillus alvei* K165 was previously shown to protect *Arabidopsis thaliana* plants against *Verticillium dahliae*. Here we show that K165 also confers inherited immune resistance to *V. dahliae.* By performing a histone acetyltransferases mutant screen, ChIP assays, and transcriptomic experiments, we were able to show that histone acetylation significantly contributes to the K165 biocontrol activity and establishment of inheritable resistance to *V. dahliae.* K165 treatment primed the expression of immune-related marker genes and the cinnamyl alcohol dehydrogenase gene *CAD3* through the function of histone acetyltransferases. Our results reveal that offspring of plants treated with K165 have primed immunity and enhanced lignification, both contributing towards the K165-mediated inherited immune resistance. Thus, our study paves the way for the use of biocontrol agents for the establishment of inheritable resistance to agronomically important pathogens.

## Teaser

A bacterial biocontrol agent confers inherited resistance to plants and protects them against an agronomically important fungal pathogen.

## Introduction

The soil-borne fungal pathogen *Verticillium dahliae* Kleb. infects >160 plant species, resulting in an estimated annual yield loss of €3 billions worldwide (Pegg and Brady, 2002). The fungus colonizes the plant vascular system causing leaf flaccidity, chlorosis, stunting, vascular discoloration in stems and flower, fruit reduction, and often necrosis (Pegg, 1981). The wide host range and the long viability of the resting structures of *V. dahliae*, in combination with the lack of effective pesticides and the scarcity of resistant germplasm, make it difficult to control the disease with conventional approaches (Fradin and Thomma, 2006).

Induced systemic resistance (ISR), often referred to as priming, has recently emerged as a complementary strategy that improves the defensive capacity of plants. Application of chemical compounds or of non-virulent strains of microbes to plants can change plant homeostasis by affecting metabolite accumulation, epigenetic marks, and transcription, all collectively contributing to faster or stronger defence responses (Mauch-Mani *et al.*, 2017). Epigenetic marks such as histone modifications and DNA methylation were shown to contribute towards the observed enhanced stress phenotypes of primed plants (Jaskiewicz *et al.*, 2011; Dowen *et al.*, 2012; Po-Wen *et al.*, 2013; Yu *et al.*, 2013; López Sánchez *et al.*, 2016; Martínez-Aguilar *et al.*, 2016).

In addition to priming, epigenetic marks have also been associated with transgenerational immune resistance. The molecular mechanism of epigenetic inheritance of disease resistance has been primarily studied by using the virulent bacteria *Pseudomonas syringae* DC3000 and β-aminobutyric acid (BABA), a chemical analogue of salicylic acid (SA). The offspring of *P. syringae* DC3000-infected and BABA-treated plants have faster and higher accumulation of transcripts of defence-related genes of the SA signalling pathway and enhanced disease resistance upon infection with *Hyaloperonospora arabidopsidis* and *P. syringae* DC3000 (Luna *et al.*, 2012; Slaughter *et al.*, 2012). This transgenerational resistance was shown to be associated with DNA methylation and increased acetylated levels of histone H3 at Lys9, at the promoter region of the SA-responsive genes in the progeny of the primed plants, while the jasmonic acid (JA)-inducible promoter of *PLANT DEFENSIN1.2* (*PDF1.2*) showed increased H3 methylation levels at Lys27 (Luna *et al.*, 2012).

In this study we investigate the inherited immune resistance induced by the biocontrol agent *Paenibacillus alvei* K165 (K165). K165 is a plant growth-promoting rhizobacterium with biocontrol activity against *V. dahliae* in glasshouse and field experiments (Tjamos *et al.*, 2004). Its mode of action against *V. dahliae* has been primarily attributed to ISR mediated by the flagellin-sensitive 2 (FLS2) receptor and SA-dependent defence pathways (Tjamos *et al.*, 2005; Gkizi *et al.*, 2016). Here we demonstrate that offspring of *Arabidopsis thaliana* plants treated with K165 are more tolerant to *V. dahliae* infections. This inherited resistance lasts for only one generation and is associated with the function of histone acetyltransferases (HATs), priming of immunity, and lignin accumulation. Our data show how the bacterial biocontrol agent K165 induce inherited immune resistance to the fungal pathogen *V. dahliae*.

## Materials and methods

### Fungal culture

*Verticillium dahliae* isolated from *Raphanus sativus* L. with known pathogenicity against *A. thaliana* plants (Tjamos *et al.*, 2005) was used in the experiments. The fungal strain was cryopreserved by freezing a conidial suspension in 25% aqueous glycerol at –80 °C (Maniatis *et al.*, 1982). Before being used, the fungus was transferred to potato dextrose agar (PDA) (Merck, Darmstadt, Germany) at 24 °C for 5 d. For the bioassays, a suspension of 10^7^ conidia ml^–1^ of distilled sterile water was prepared from a culture grown for 5 d at 24 °C in sucrose sodium nitrate liquid medium (Sinha and Wood, 1968).

### Bacterial culture

A K165 rifampicin-resistant mutant (Tjamos *et al.*, 2004) was used throughout the experiments. The K165 strain was cryopreserved by freezing a suspension of 1×10^8^ colony-forming units (cfu) ml^–1^ in 25% aqueous glycerol at –80 °C (Maniatis *et al.*, 1982). Before being used, K165 was transferred to nutrient broth agar plus glycerol (NAG) at 28 °C for 2 d. For the bioassays, bacterial cells were prepared in NAG in an orbital incubator at 180 rpm and 28 °C for 2 d. Suspensions were centrifuged at 5600 *g* at 20 °C for 10 min and resuspended in sterile distilled water before treatment of the plants.

### Plant material and growth conditions

The *A. thaliana* ecotype Columbia (Col-0) was used as the wild type. The mutant lines *gnc5* (*hag1*), *hag2*, *hag4*, *hag5*, *hac1*, *hac4*, *hac5*, *hac12*, and *cad3* in the Col-0 ecotype background were used (Supplementary Table S1). Seeds were stored at 4 °C. For the bioassays, *A. thaliana* seeds were sown in pots (9×9×10 cm) containing a pasteurized soil mix of humus and perlite (3:1) and were maintained at 25 °C with a 12 h photoperiod at 60–70% relative humidity in a controlled-environment growth chamber. After 10 d, the plants were planted singly in plastic pots with ~80 cm^3^ of pasteurized soil mix of humus and perlite (3:1).

### Generation of first and second progeny of K165-primed plants

At the stage of four true leaves (~18 d old), plants (Col-0, and *hag*, *hac*, and *cad* mutants) were inoculated with K165 by root drenching, using 10 ml of 10^8^ cfu ml^–1^, or mock inoculated with sterile distilled water. Seeds were collected and air dried. In order to exclude seed infection by K165, the seeds were placed on PDA plates at 28 °C for 10 d. Plants grown from the seeds of those parent plants (P0) were designated as P1. Next P1 plants were grown and seeds were collected from those plants. Plants grown from the seeds of P1 plants were designated as P2.

### *V. dahliae* bioassays in *A. thaliana*

At the stage of four true leaves (~18 d old), plants were challenge-inoculated with *V. dahliae* by root drenching with 10 ml of a suspension of 10^7^ conidia ml^–1^ of sterile distilled water (Tjamos *et al.*, 2005). Control plants were mock inoculated with 10 ml of sterile distilled water.

Disease scoring started at 9 days post-inoculation (dpi) and was performed every 2 d until 19 dpi.The disease severity at each observation was calculated from the number of leaves that showed wilting as a percentage of the total number of leaves of each plant. Disease ratings were plotted over time to generate disease progression curves, and the area under the disease progress curve (AUDPC) was calculated by the trapezoidal integration method (Campbell and Madden, 1990). Disease was expressed as a percentage of the maximum possible area for the whole period of the experiment, which is referred to as the relative AUDPC. The experiment was repeated three times with 10 plants per treatment and plant genotype (a total of 30 plants).

### K165–*V. dahliae* bioassays in *A. thaliana*

At the stage of four true leaves (~18 d old), plants were inoculated with K165 by root drenching, using 10 ml of 10^8^ cfu ml^–1^. Control plants were mock-inoculated with 10 ml of sterile distilled water. After 5 d, plants were challenge-inoculated with *V. dahliae* by root drenching with 10 ml of a suspension of 10^7^ conidia ml^–1^; mock-treated plants were inoculated with 10 ml of sterile distilled water (Tjamos *et al.*, 2005). The disease scoring and analysis was performed as described above.

### DNA extraction and qPCR fungal quantification

K165 was inoculated into 4-week-old Arabidopsis plants 5 d prior to infection with *V. dahliae*. The aerial parts of 30 plants per treatment were harvested at 19 dpi with the fungus (end of the pathogenicity experiments), for quantitative PCR (qPCR) quantification of the *V. dahliae* endophytic levels. In brief, the aerial parts of the plants were cut at soil level, pooled in groups of five, rinsed with sterile distilled water, and ground to a fine powder, using an autoclaved mortar and pestle in the presence of liquid nitrogen.

Total DNA was isolated according to Dellaporta *et al.* (1983) and the concentration was estimated by using NanoDrop UV spectrophotometry. qPCR assays for the quantification of *V. dahliae* were conducted as described previously by Fradin *et al.* (2011), using a pair of primers (Supplementary Table S2) designed for the internal transcribed spacer 1 (ITS1) and ITS2 regions of the 5.8S rRNA gene (Z29511) of *V. dahliae.* qPCRs were performed in an Applied Biosystems StepOne Plus thermocycler and, for the amplification reactions, FastGene IC Green qPCR universal mix (NIPPON Genetics EUROPE GmbH) was used. The results were analysed with StepOne v.2.3 qPCR software. For sample calibration, the Arabidopsis gene *At4g26410*, previously described as a stable reference gene (Czechowski *et al.*, 2005), was targeted. PCR cycling started with an initial step of denaturation at 95 °C for 2 min, followed by 40 cycles of 95 °C for 5 s and 60 °C for 30 s. PCR efficiency for each amplicon was calculated by employing the linear regression method on log (fluorescence) per cycle number data, using Lin-Reg PCR software (Ramakers *et al.*, 2003). Five biological repeats were conducted with six plants per treatment and per repeat (a total of 30 plants per treatment). The absence of non-specific products and primer dimers was confirmed by the analysis of melting curves.

### Microarray experiment

Gene expression profiling was performed using Affymetrix GeneChip® AraGene-1_0-st (Affymetrix United Kingdom Ltd, High Wycombe, UK) containing 28 583 genes. RNA quantity and quality were determined by measurement of the concentration with absorbance at 260 nm and 280 nm (NanoDrop 2000c; Thermo Fisher Scientific, Bonn, Germany) and by means of an Agilent 2100 Bioanalyzer with an RNA 6000 Nano kit and 2100 Expert software (version B.02.07) (all Agilent Technologies Deutschland GmbH, Boeblingen, Germany) at the Core Facility Genomics of the Medical Faculty Münster. The fragmented and biotinylated DNA targets were prepared according to the standard Affymetrix WT PLUS Reagent Kit protocol (Affymetrix GeneChip® WT PLUS Reagent Kit, 902280) from 100 ng of total RNA starting material and 5.5 µg of cDNA intermediate product. DNA targets were hybridized for 17 h at 45 °C on AraGene-1_0-st Arrays. GeneChips were washed and stained in the Affymetrix Fluidics Station 450 according to the standard GeneChip Expression Wash, Stain and Scan protocol (Affymetrix GeneChip Wash, Stain and Scan Kit, 900720). The GeneChips were scanned using the Affymetrix 3000 7G scanner. For microarray data analysis, the Affymetrix Expression Console and Transcriptome Analysis Console was used. The robust multiarray averaging method was applied for background correction, normalization, and probe summarization. Gene expression differences were determined by applying an ANOVA.

### ChIP assays coupled to qPCR

The direct and inheritable effect of K165 on the acetylation levels of Lys9 and Lys14 at histone 3 was determined in wild-type P0 treated with K165, and P1 and P2 plants. For this purpose, seeds of P0, P1, and P2 were placed on plates containing half-strength MS medium (Murashige and Skoog, 1962), supplemented with 10 g l^–1^ sucrose (Sigma Aldrich, St Louis, MO, USA), 5 g l^–1^ phytagel (Sigma Aldrich), and 25 mg l^–1^ nystatin (Sigma Aldrich), and allowed to grow in a controlled-environment chamber with a 12 h photoperiod at 22 °C. Two weeks later, 100 µl of K165 (1×10^8^ cfu ml^–1^, prepared as described previously) were applied per P0 plant (except for the mock-inoculated P0). After 24 h, the plants of the different treatments were sampled for ChIP analysis (Carles and Fletcher, 2009). Briefly, 2 g of fresh tissue per sample were cross-linked through vacuum infiltration (85000 Pa, 10 min vacuum during three rounds) in cross-linking solution [40 mM sucrose, 1 mM phenylmethylsulfonyl fluoride (PMSF), 10 mM Tris–HCl pH 8.00, 1 mM EDTA, and 1% formaldehyde]. Afterwards, freshly prepared 2 M glycine was added to the cross-linking solution to a final concentration of 125 mM and vacuum infiltrated for 5 min. Cross-linked tissue was pulverized into a fine powder and resuspended in cold Honda Buffer containing 440 mM sucrose, 20 mM HEPES KOH pH 7.4, 10 mM MgCl_2_, 1.25% Ficoll (GE Healthcare), 2.5% dextran T40 (Sigma), 5 mM DTT, 1 mM PMSF, 0.5% Triton X-100, and 0.2% plant protease inihibitor cocktail (Sigma) at a ratio of 2:1, and filtered through two layers of Miracloth (Millipore). Samples were incubated in a rotating wheel at 4 °C for 15 min and centrifugated at 3000 *g* for 30 min at 4 °C. Precipitated nuclei were resuspended in 1 ml of Honda Buffer and washed several times. Nuclei were ruptured using lysis buffer (50 mM Tris–HCl pH 8, 1% SDS, 10 mM EDTA 1 mM PMSF. and 0.2% plant protease inhibitor cocktail) and sonicated using the Bioruptor (Diagenode) (high power, 3×30 s on, 60 s off) to break the chromatin. Samples were centrifuged in a bench-top centrifuge at maximum speed for 30 min at 4 °C to break the nuclei. A 10 μl aliquot of sonicated chromatin per sample was kept as input and 25 μg of nuclear extracts were diluted in ChIP-dilution buffer (1.2 mM EDTA, 1.67 mM Tris–HCl pH 8, 167 mM NaCl, 1.1% Triton X-100, 0.2% plant protease inhibitor cocktail), probed with the antibodies antiH3K9K14ac (06-599, Millipore) and anti-H3 (ab1791, Abcam), and incubated overnight in a rotating wheel at 4 °C. Dynabeads™ Protein A beads (ThermoFisher) were added to each sample and incubated for 2 h in the rotating wheel at 4 °C. Beads were washed with binding buffer (150 mM NaCl, 20 mM Tris–HCl pH 8, 2 mM EDTA, 1% Triton X-100, 0.1% SDS, 1 mM PMSF, and 0.2% plant protease inhibitor cocktail) and immunoprecipitated chromatin was eluted in glycine elution buffer (0.1 M glycine, 0.5 M NaCl, 0.05% Tween-20, pH 2.8). Eluted chromatin and input samples were incubated with 1 μl of RNase A (10 mg ml^–1^) for 15 min at 37 °C and 3 μl of proteinase K at 37 °C overnight. DNA was purified using the MinElute Kit (Quiagen) and quantified using a spectrophotometer (NanoDrop, ThermoFisher). Real-time PCR was performed on immunoprecipitated DNA samples, and relative enrichments were calculated as the percentage of the obtained values for the immunoprecipitated and input fractions. Two sets of primers were used for each gene of interest, one of them targeting the promoter and the other the ORF (Supplementary Table S3).

### RNA isolation and qPCR determination of transcript levels

Aerial tissues were collected for transcriptomic analysis. Five repeats were conducted per treatment with five plants per treatment and repeat. P0 and K165-treated P0 and P1 plants were inoculated with *V. dahliae* and tissue was harvested, immediately frozen in liquid nitrogen, and stored at –80 °C at 3 and 7 dpi. For each sample, total RNA was extracted from 100 mg of tissue ground with liquid nitrogen, using TRIzol reagent (Invitrogen, Paisley, UK) according to the manufacturer’s instructions. The RNA samples were treated with DNase I (Invitrogen) to eliminate traces of contaminating genomic DNA. The RNA concentration was measured on a Nanodrop ND-1000 spectrophotometer (Saveen Werner, Malmö, Sweden). First-strand cDNA was synthesized, using the Prime Script RT reagent kit (TAKARA BIO INC.) following the manufacturer’s procedure. For the amplification of *PR1* (At2g19990), *PDF1.2* (At5g44420), and *NPR1* (At1g64280) with real-time PCR, primer sets designed by Trusov *et al.* (2009) and Pantelides *et al.* (2010) were used (Supplementary Table S4). For the amplification of *CAD4* (*At3g19450*), a specific primer pair was designed in Primer-BLAST (Ye. *et al*., 2012) and, for the amplification of *CAD3* (*At2g21890*), a primer set published by Raes *et al.* (2003) was used.

Normalization of gene expression, PCR efficiency, absence of non-specific products and primer dimers, and data analysis were performed as described above in the qPCR fungal quantification procedure. Under the experimental infection conditions, the expression of *At4g26410* (Czechowski *et al.*, 2005), used for normalization of gene expression, was stable between treatments, with cycle threshold values of ~19.5.

### Lignin quantification

Aerial tissues were collected for lignin quantification. Five repeats were conducted per treatment, with five plants per treatment and repeat, at 3 and 7 dpi with *V. dahliae* and were immediately frozen in liquid nitrogen and stored at –80 °C. For each sample, lignin was extracted according to Schenk and Schikora (2015). In brief, the soluble phenolics were extracted with 80% methanol and the cell wall-bound phenolics were hydrolysed (alkaline hydrolysis) and dissolved in ethyl acetate. Lignin was extracted by binding to the lingo-thioglycolic acid complex. The lignin complex was dissolved in NaOH and the absorbance was measured at 340 nm. A standard curve for lignin was generated, by using lignin, alkali (Sigma-Aldrich; 370959).

## Results

### K165 confers inherited immune resistance

**Fig. 1.**
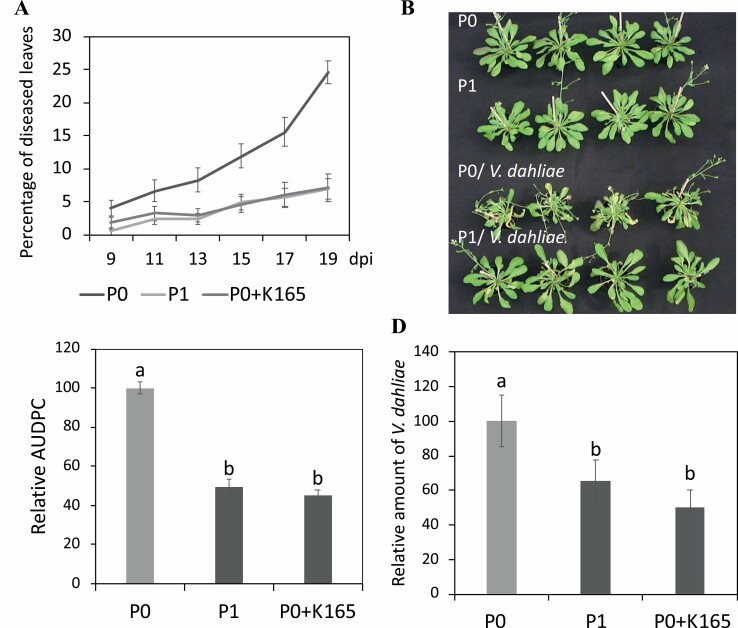
K165-mediated inherited resistance to *V. dahliae***.** (A–C) Immunity phenotypes of untreated controls plants (P0), K165-treated P0 plants (P0+K165), and offspring of K165-treated P0 plants (P1) against *V. dahliae*. The immunity phenotypes are expressed as the percentage of diseased leaves starting at 9 days post-inoculation (dpi), and were assessed every 2 d until 19 dpi. The photograph showing visible Verticillium wilt symptoms was taken at 19 dpi with *V. dahliae*. The area under the disease progress curve (AUDPC) was calculated as a percentage of the disease of the P0 plants. The experiments were repeated three times (*n*=10) with similar results. (D) The relative endophytic level of *V. dahliae* DNA in P0, P0+K165, and P1 plants was determined at 19 dpi as a percentage of the fungal level in P0 plants. The experiment was repeated three times (*n*=6). In all experiments, error bars represent ±SE and different letters denote significance differences according to ANOVA, followed by Tukey’s multiple range test, *P*<0.05.

In order to determine whether the K165 biocontrol activity against *V. dahliae* extends to the offspring of plants treated with K165, we performed a series of pathogenicity experiments. Control plants (P0), plants treated with K165 (P0+K165), offspring of plants treated with K165 (P1), and offspring of P1 plants (P2) were infected with *V. dahliae.* Verticillium symptoms were recorded from 9 to 19 dpi, in the form of wilting. Disease severity progressed faster in the P0 plants compared with P0+K165 and P1 plants. At 19 dpi, the disease severity of the *V. dahliae-*infected P0 plants was 25%, while in the P1 plants it was 5% (Fig. 1A, B). Furthermore, the overall amount of disease, AUDPC, was reduced by 50% in P1 and P0+K165 plants compared with P0 plants (Fig. 1C) and the detected endophytic relative level of *V. dahliae* DNA in P1 and P0+K165 plants was significantly lower than in P0 plants (Fig. 1D). In contrast, P2 plants were as susceptible as P0 plants to *V. dahliae* and we did not observe any significant difference between them throughout the experimental time period (Supplementary Fig. S1).

Collectively, the pathogenicity experiments revealed that the K165 biocontrol activity against *V. dahliae* extends to the offspring P1 plants but did not persist into the subsequent stress-free generation (P2 plants). Therefore, K165-mediated resistance is inheritable but not transgenerational.

### Histone acetyltransferases contribute to the K165-mediated biocontrol activity and the establishment of the inherited immune resistance to *V. dahliae*

Histone marks and particularly histone 3 Lys9 and Lys14 acetylation (H3K9K14 acetylation) have been previously associated with priming of defences (Jaskiewicz *et al.*, 2011) and transgenerational immune resistance (Luna *et al.*, 2012). Therefore, we investigated the potential role of HATs and histone 3 acetylation in K165-mediated biocontrol activity and establishment of inherited immune resistance.

**Fig. 2.**
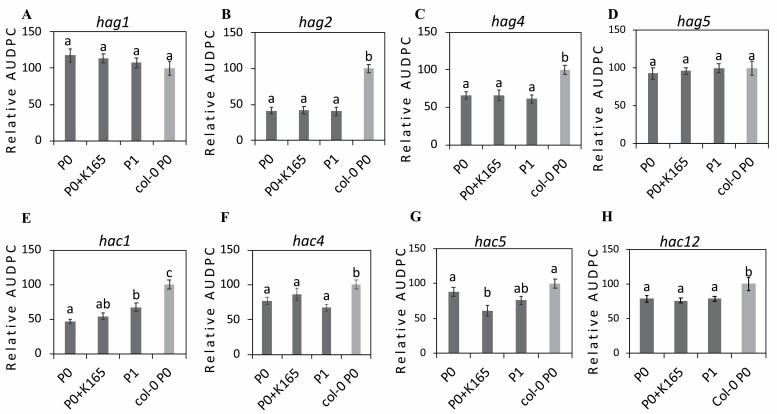
Histone acetyltransferases contribute to the K165-mediated biocontrol activity and the establishment of inherited resistance to *V. dahliae*. (A–H) Immunity phenotypes of untreated controls plants (P0), K165-treated P0 plants (P0+K165), and offspring of K165-treated P0 plants (P1) of the indicated GNAT (A and B), MYST (C and D), and CBP (E–H) histone acetyltransferase mutants against *V. dahliae.* Col-0 P0 plants were used as control. Relative disease levels were calculated as the area under the disease progress curve (AUDPC) and expressed as a percentage of the disease of the Col-0 P0 plants. Different letters denote significance differences according to ANOVA, followed by Tukey’s multiple range test, *P*<0.05. Experiments were repeated three times (*n*=10), and error bars represent ±SE.

Plant HATs are typically classified into four main families (GNAT, MYST, CBP, and TAFII250) on the basis of homology with other eukaryotic HATs. P0 and P1 homozygous T-DNA insertion mutants of eight (four *HAG* genes belonging to the GNAT and MYST families and the four *HAC* genes belonging to the CBP family) out of the 12 Arabidopsis HATs (Supplementary Table S1) were assessed for their immunity phenotypes against *V. dahliae* (Fig. 2). Our analysis revealed that P0 plants of the *hag2*, *hag4, hac1*, *hac4*, and *hac12* mutants had enhanced tolerance against *V. dahliae* (Fig. 2; Supplementary Figs S2, S3). From the eight HAT mutants tested, application of K165 to P0 mutants only partially protected *hac5* plants against *V. dahliae*, but these results were not supported by the relative endophytic level of the *V. dahliae* (Supplementary Fig. S4). In addition, none of the P1 mutants exhibit the 50% reduction of AUDPC observed in the control Col-0 plants (Figs 1C, 2A–H). Therefore, both the K165-mediated biocontrol activity and the establishment of the inheritable resistance to *V. dahliae* require the function of HATs, but the exact role of each HAT is unclear.

To ascertain the involvement of histone 3 acetylation in the biocontrol activity of K165 and the establishment of inherited resistance, we evaluated the H3K9K14 acetylation levels in the promoters and the ORFs of the NONEXPRESSOR OF PATHOGENESIS-RELATED GENES 1 (*NPR1*), PATHOGENESIS-RELATED PROTEIN 1 (*PR1*), *PDF1.2*, and CINNAMYL ALCOHOL DEHYDROGENASE 3 (*CAD3*) and 4 (*CAD4*) genes.

The immune-related marker genes of SA (*NPR1* and *PR1)* and JA (*PDF1.2*) were selected based on our previously work indicating the involvement of both pathways in K165-induced resistance (Gkizi *et al.*, 2016). The genes encoding the two cinnamyl alcohol dehydrogenases, *CAD3* and *CAD4*, were selected based on their role in lignin biosynthesis and a preliminary microarray experiment indicating that K165 affects the phenylpropanoid biosynthesis pathway and, in particular, the expression of these two genes (Supplementary Fig. S5; Gkizi, 2019).

**Fig. 3.**
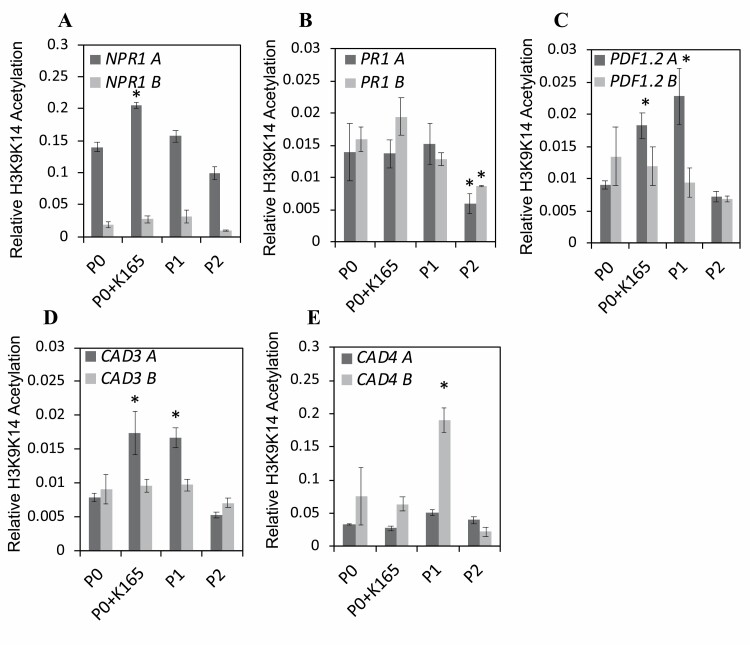
K165-mediated inheritable increase of H3K9K14 acetylation in promoters and gene bodies of defence and lignin biosynthesis genes. Relative H3K9K14 acetylation levels of *NPR1* (A), *PR1* (B), *PDF1.2* (C), *CAD3* (D), and *CAD4* (E) genes in untreated controls plants (P0), K165-treated P0 plants (P0+K165), offspring of K165-treated P0 plants (P1), and offspring of P1 plants (P2). Promoter (A) and gene body (B) regions of the indicated genes were analysed by ChIP-qPCR using α-H3 and α-H3K9K14 acetylation antibodies. Levels of H3K9K14 acetylation for each region were calculated relative to total H3 immunoprecipitated, and normalized against the H3K9K14ac levels of the housekeeping gene Actin. Data represent the means of three ChIp-qPCRs from three independent replicates (*n*=100). Experiments were performed three times with similar results, error bars represent ±SE, and asterisks (*) denote significance differences from P0 acetylation levels using *t*-test, *P*<0.05.

The histone H3K9K14 acetylation levels of most tested genes in P0 and P2 plants were indistinguishable. In contrast, there were significant differences in H3K9K14 acetylation levels among P0, K165-treated P0, and P1 plants (Fig. 3). H3K9K14 acetylation levels were higher in the *NPR1* promoter of the K165-treated plants in comparison with P0 plants (Fig. 3A). In contrast, the acetylation levels of the *PR1* promoter were indistinguishable among the P0, K165-treated P0, and P1 plants, and lower in P2 plants (Fig. 3B). Similar to *NPR1*, the *PDF1.2* promoter of the K165-treated P0 plants had higher H3K9K14 acetylation in comparison with P0 plants, but unlike *NPR1* the acetylation levels of the *PDF1.2* promoter remained higher in P1 plants (Fig. 3C). In contrast, H3K9K14 acetylation levels of the ORFs of *NPR1* and *PDF1.2* genes did not significantly differ between P0, K165-treated P0, P1, or P2 plants (Fig. 3A–C). Similar to *PDF1.2*, the *CAD3* promoter, but not the ORF, displayed higher H3K9K14 acetylation levels both in K165-treated P0 plants and in their offspring (P1) in comparison with P0 plants (Fig. 3D). Finally, the ORF of the *CAD4* gene in P1 plants was found to be hyperacetylated compared with P0, K165-treated P0, and P2 plants (Fig. 3E). Thus, the K165 treatment enhances H3K9K14 acetylation levels in the promoters of *NPR1*, *PDF1.2*, and *CAD3* genes, but only in the case of *PDF1.2* and *CAD3* do the higher acetylation levels persist in P1 plants and none of these differences in acetylation was maintained in P2 plants.

**Fig. 4.**
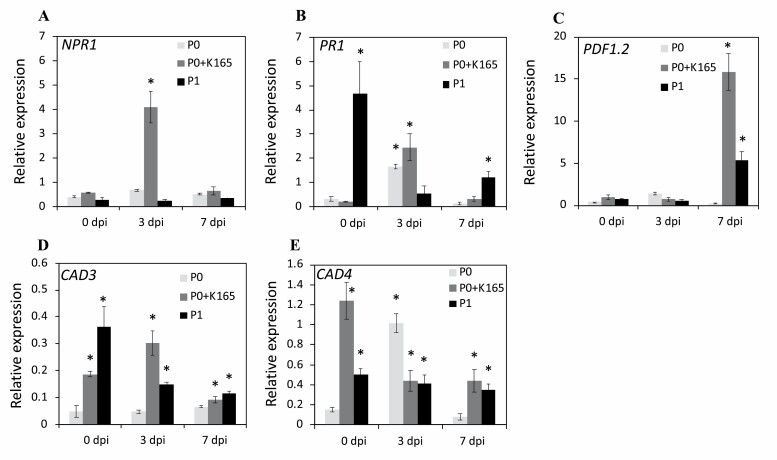
K165-mediated transcription of defence and lignin biosynthesis genes. Gene expression levels of *NPR1* (A), *PR1* (B), *PDF1.2* (C), *CAD3* (D), and *CAD4* (E) in untreated control plants (P0), K165-treated P0 plants (P0+K165), and offspring of K165-treated P0 plants (P1) at 0, 3, and 7 days post-inoculation (dpi) with *V. dahliae*. Expression levels of the indicated genes relative to a housekeeping gene (*AT4G26410*) are shown. Values are means ±SE, *n*=10. Experiments were performed three times with similar results, and asterisks (*) denote significance differences from P0 at 0 dpi in relative expression levels using *t*-test, *P*<0.05.

In order to correlate the K165-induced changes in H3K9K14 acetylation levels with transcriptional regulation, we quantified the expression levels of *NPR1*, *PR1*, *PDF1.2*, *CAD3*, and *CAD4* genes in adult (4-week-old) plants before and after infections with *V. dahliae* (Fig. 4) and in 2-week-old seedlings (Supplementary Fig. S6). Consistent with the higher H3K9K14 acetylation levels of the *NPR1* promoter (Fig. 3A), *NPR1* expression was higher in K165-treated P0 adult plants at 3 dpi with *V. dahliae* and in K165-treated P0 and P1 seedlings in comparison with P0 control plants (Fig. 4A; Supplementary Fig. S6A). In contrast, the expression levels of *PR1* in adult plants (Fig. 4B) and seedlings (Supplementary Fig. S6B) was not correlated with the histone acetylation levels of its promoter since no increase was observed in the H3K9K14 acetylation levels (Fig. 3B). Similar to *NPR1*, the higher H3K9K14 acetylation levels of the *PDF1.2* promoter of K165-treated P0 and P1 plants were corelated with *PDF1.2* expression levels in adult plants at 7 dpi with *V. dahliae* (Fig. 4C) and in seedlings (Supplementary Fig. S6C). Furthermore, the higher H3K9K14 acetylation levels of the *CAD3* promoter of K165-treated P0 and P1 plants were correlated with *CAD3* expression levels in adult plants but not in seedlings, suggesting a further developmental regulation of *CAD3* expression (Fig. 4D; Supplementary Fig. S6D). Finally, the higher H3K9K14 acetylation levels in the ORF of *CAD4* in P1 plants were not correlated with *CAD4* expression levels in adult plants and seedlings (Fig. 4E; Supplementary Fig. S6E).

Thus, at least in the case of *NPR1*, *PDF1.2*, and *CAD3* genes, there is an association between the K165-induced acetylation and transcription levels which further support the hypothesis that histone acetylation contributes to the establishment of the K165-mediated inheritable resistance to *V. dahliae*.

### Lignin levels contribute to the K165 biocontrol activity against *V. dahliae*

**Fig. 5.**
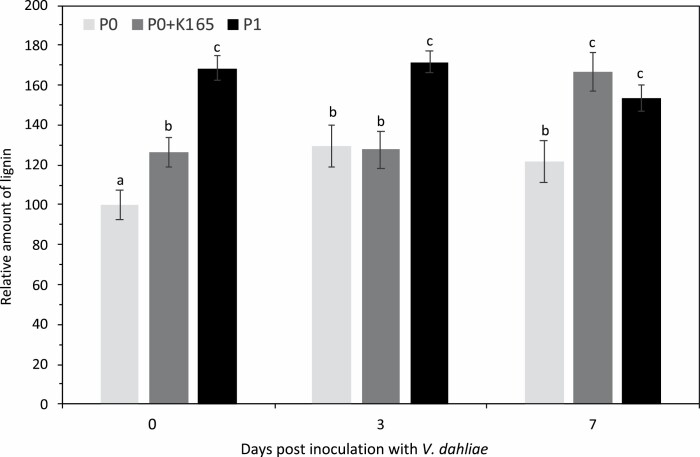
Lignin accumulation contributes to K165-mediated inherited resistance to *V. dahliae*. Relative lignin levels in untreated control plants (P0), K165-treated P0 plants (P0+K165), and offspring of K165-treated P0 plants (P1) at 0, 3, and 7 days post-inoculation with *V. dahliae.* Relative lignin levels were calculated as a percentage of the lignin levels of the P0 plants. Different letters denote significance differences according to ANOVA, followed by Tukey’s multiple range test, *P*<0.05. All experiments were repeated five times (*n*=5) with similar results, and error bars represent ±SE.

**Fig. 6.**
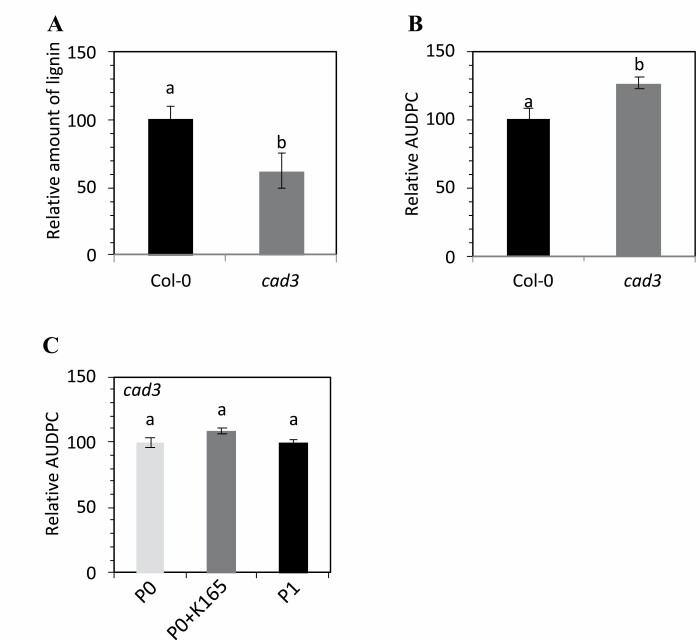
*CAD3* contributes to K165-mediated inherited resistance to *V. dahliae.* (A) Relative lignin levels of offspring of K165-treated plants (P1) of Col-0 and *cad3* plants. Relative lignin levels were calculated as a percentage of the lignin levels of the Col-0 P1 plants. All experiments were repeated five times (*n*=5). (B) Immunity phenotype of Col-0 and *cad3* mutant plants against *V. dahliae.* Relative disease levels were calculated as the area under the disease progress curve (AUDPC) and expressed as a percentage of the disease of the Col-0 plants. Experiments were repeated three times (*n*=10). (C) Immunity phenotypes of *cad3* untreated plants (P0), K165-treated P0 (P0+K165), and P1 plants against *V. dahliae.* Relative disease levels were calculated as in (B). For all experiments, different letters denote significance differences using *t*-test, *P*<0.05, and error bars represent ±SE.

Our experiments revealed a K165-induced hyperacetylation and expression of the cinnamyl alcohol dehydrogenase gene *CAD3* (Figs 3D, 4D). Given the role of cinnamyl alcohol dehydrogenases in lignin biosynthesis, we next investigated the role of lignin in the K165-mediated biocontrol activity. Treatment with K165 increased the lignin levels of P0 plants by 20%, and subsequent inoculation with *V. dahliae* led to higher lignin levels at 7 dpi (Fig. 5). The lignin levels in P1 plants were ~60% higher in comparison with P0 plants both before and after infections with *V. dahliae* (Fig. 5), indicating that lignin biosynthesis contributes to the K165-mediated establishment of inherited resistance to *V. dahliae*. In contrast, the lignin levels of the P1 *cad3* plants were 40% lower in comparison with P1 Col-0 plants (Fig. 6A), but the lignin levels between the P0 Col-0 and *cad3* plants were indistinguishable (Supplementary Fig. S7).

In order to fully understand the role of *CAD3* in the K165-mediated biocontrol activity and establishment of inherited resistance, we performed additional pathogenicity experiments. Our experiments showed that P0 *cad3* plants are more susceptible to *V. dahliae* (Fig. 6B); application of K165 failed to protect them against *V. dahliae* and to enhance the resistance of P1 *cad3* plants against the fungus (Fig. 6C). Therefore, *CAD3*-mediated lignification is required for resistance to *V. dahliae* and the K165-mediated enhanced lignification and biocontrol activity against *V. dahliae*.

**Fig. 7.**
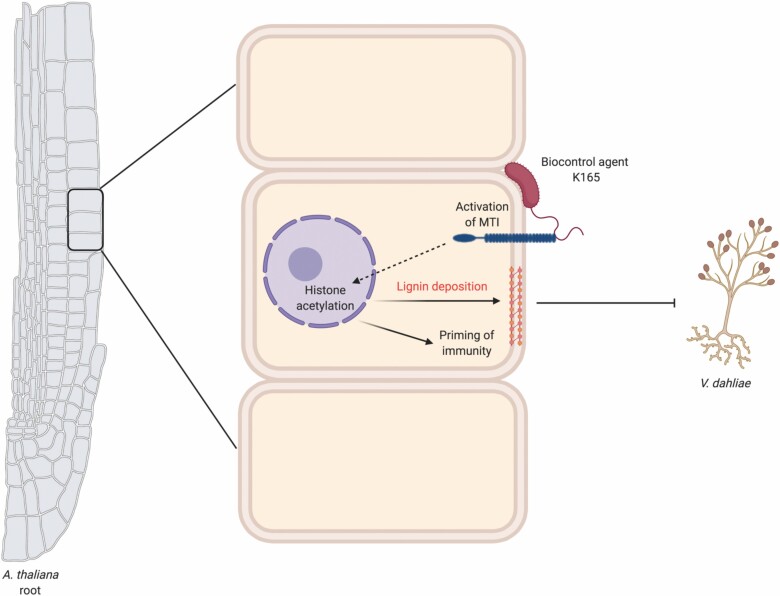
Model showing the proposed function of K165-mediated plant protection. The K165-mediated biocontrol activity and establishment of inherited resistance to *V. dahliae* depends on the activation of MAMP (microbe associated molecular pattern)-triggered immunity (MTI) and the subsequent priming of immunity and lignin accumulation through the function of histone acetyltransferases. The model was created using BioRender.com.

Collectively our data demonstrate that K165 biocontrol activity and establishment of inherited resistance are both mediated by histone acetylation that primes the expression of immunity-related genes and lignin accumulation (Fig. 7).

## Discussion

The effectiveness of several biocontrol agents, K165 included, against *V. dahliae* in plants is well documented (Berg *et al.*, 1994; Mercado-Blanco *et al.*, 2004; Tjamos *et al.*, 2004), but is not clear if their biocontrol activity can be extended to offspring plants. Our study provides evidence about the role of histone acetylation in the establishment of biocontrol agent-induced inherited resistance to *V. dahliae*. The first progeny (P1) of plants treated with the biocontrol strain K165 had enhanced tolerance to *V. dahliae* (Fig. 1), but this enhanced immunity phenotype did not persist to the second progeny (P2) plants (Supplementary Fig. S1). The mechanism underlying the establishment of the K165-mediated inheritable resistance to *V. dahliae* was further investigated using HAT mutant plants. From the eight *HAT* gene mutants used in this study, the four *HAG* genes belong to the GNAT and MYST families, and the four *HAC* genes to the CBP family. In general, these enzymes modify more than one lysine residue, but some of them have been described to show specificity for particular residues and are known to control multiple plant phenotypes (Allis *et al.*, 2007; Earley *et al.*, 2007; Desvoyes *et al.*, 2010; Singh *et al.*, 2014). The observed enhanced tolerance phenotype of multiple HAT mutants against *V. dahliae* prior to K165 treatment (Fig. 2) adds to a growing body of publications reporting the role of HATs in plant immunity (Singh *et al.*, 2014; Kim *et al.*, 2020). The application of K165 in the *hag* and *hac* P0 plants failed to reduce the symptoms caused by *V. dahliae* and to confer inheritable resistance. Therefore, it is clear that multiple *HAC* and *HAG* genes are involved in the K165-mediated plant protection against *V. dahliae*, but their exact role is unclear.

Similar cases of bacterial-induced inherited resistance have been previously reported (Luna *et al.*, 2012; Rasmann *et al.*, 2012; Slaughter *et al.*, 2012). In the study of Luna *et al.* (2012), Arabidopsis plants were primed by repeated inoculations with *P. syringae*, and the primed state was passed on to the next generation and was sustained over one stress-free generation, making plants more resistant to *H. arabidopsidis* and *P. syringae*. The progeny of the *P. syringae*-primed plants had increased acetylated levels of Lys9 of histone 3 at the promoter region of the SA-responsive genes. In accordance, K165 treatment resulted in increased acetylation levels of histone 3 at Lys9 and Lys14 at the promoter region of immune-related genes (Fig. 3). Our mutant screen and ChIP experiments indicate that histone acetylation plays a significant role in K165-mediated biocontrol activity and establishment of inherited resistance. In addition, our data also show that K165 treatment resulted in higher *PR1* expression that was not correlated with the histone acetylation levels of its promoter, suggesting the involvement of additional mechanisms in K165-mediated plant protection. However, unlike the case of *P. syringae*-primed plants, the K165-mediated inherited resistance is not sustained over one stress-free generation and therefore cannot be classified as transgenerational resistance. It is important to note that DNA methylation is the most plausible mechanism by which the K165-mediated inherited resistance to *V. dahliae* is extended to offspring plants (Luna and Ton, 2012; Stassen *et al.*, 2018); therefore, histone acetylation is most probably related to the establishment of the inherited resistance.

An interesting conclusion of our results is that the expression levels of both SA (*NPR1* and *PR1*) and JA (*PDF1.2*) immune-related marker genes were enhanced by K165 treatment (Fig. 4). It is well established that there is a crosstalk between the SA- and ethylene (ET)/JA-dependent signalling pathways. In most documented examples, the SA and ET/JA signalling pathways interact antagonistically (Koornneef and Pieterse, 2008). However, in our results, the up-regulation of *NPR1*, *PR1*, and *PDF1.2* in the K165-treated plants suggests the simultaneous activation of the SA- and ET/JA-dependent plant defences. Similar results have been previously obtained by Niu *et al.* (2011) when they studied the interaction of *Bacillus cereus AR156* with *P. syringae*. According to Jiang *et al.* (2016), the simultaneous activation of the SA and ET/JA signalling pathways is modulated via the transcription factor genes *WRKY11* and *WRKY70*, and leads to an additive effect on the level of induced protection. Furthermore, Betsuyaku *et al.* (2018) shed further light on the simultaneous activation of the SA and ET/JA signalling pathways by examining the spatiotemporal dynamics of defence-related promoter activities during effector-triggered immunity in *A. thaliana*. It was observed that the SA and JA pathways are activated in distinct concentric domains; inner SA and outer JA domains around the hypersensitive reaction cell death area, explaining the simultaneous activation of the SA and ET/JA pathways in terms of a spatial differentiation. The SA pathway is important for defence against biotrophs, while the ET/JA pathway is essential against necrotrophs. Therefore, the SA pathway may act against the initial biotrophic mode of lifestyle of *V. dahliae* and the ET/JA pathway against the later necrotrophic stages of infection.

Lignin is considered as an important barrier that *V. dahliae* has to overcome in order to infect a plant. It was previously reported that the resistance levels of cotton varieties to *V. dahliae* are positively correlated with their lignin content (Xu *et al.*, 2011), *Verticillium* infection triggers *de novo* xylem formation in Arabidopsis (Reusche *et al.*, 2012), *V. dahliae* colonization in pepper is limited by lignin accumulation in cell walls (Novo *et al.*, 2017), and the *R* gene Ve provokes a resistance response to *V. dahliae* in tomato by increasing lignin (Gayoso *et al.*, 2010). Several lines of evidence indicate that lignin levels also contribute to the K165-mediated biocontrol activity and establishment of inherited resistance to *V. dahliae.* First, the expression of the cinnamyl alcohol dehydrogenase gene *CAD3* was higher in K165-treated P0 and their offspring P1 plants than in P0 plants, and in both cases the expression levels were positively correlated with histone 3 Lys9 and Lys14 acetylation of the *CAD3* promoter (Figs 3, 4). Second, K165 failed to protect *cad3* mutant plants and their offspring P1 plants against *V. dahliae* (Fig. 6C). Finally, K165-treated P0 and P1 plants had higher lignin levels than P0 plants, and *V. dahliae* infection enhanced lignin levels of P0 and K165-treated P0 plants (Fig. 5). Therefore, the K165 treatment contributes to K165-mediated biocontrol activity by enhancing fungal-induced lignification, while the pre-formed higher lignin levels of P1 plants contribute to K165-mediated inheritable resistance. Cinnamyl alcohol dehydrogenases catalyse the reduction of cinnamaldehyde lignin precursors into cinnamyl alcohols (Boudet, 2000), and in Arabidopsis nine genes have been annotated as encoding cinnamyl alcohol dehydrogenases (Costa *et al.*, 2003). *CAD3*, despite having mild catalytic activity, is not expressed in lignifying tissues and it is therefore assumed that it does not contribute to the structural lignification during development (Kim *et al.*, 2004, 2007). Consistent with this observation, P0 Col-0 plants and *cad3* mutants have indistinguishable levels of lignin (Supplementary Fig. S7A). In contrast, P1 *cad3* plants have significantly lower levels of lignin than Col-0 P1 plants (Fig. 6A). Similarly, the cinnamyl alcohol dehydrogenase CAD7 was recently shown to regulate plant immunity without contributing to structural lignification (Li *et al.*, 2019). Unlike CAD7, CAD3 is a positive regulator of immunity and contributes to K165-mediated lignification. Thus, the two cinnamyl alcohol dehydrogenases appear to have opposite roles in plant immunity.

The mechanism(s) underlying K165-mediated biocontrol activity and establishment of inherited resistance is not completely understood. We have previously reported that K165 biocontrol activity is mediated by FLS2- and SA-dependent pathways. In this study, we were able to demonstrate that both the K165-mediated biocontrol activity and the establishment of inherited resistance are dependent on histone acetylation, which in turn simultaneously regulates the priming of immunity and lignin accumulation (Fig. 7). Thus, our study paves the way for the use of biocontrol agents for the establishment of inheritable resistance to agronomically important pathogens.

## Supplementary data

The following supplementary data are available at *JXB* online.

Table S1. Mutant lines of histone acetyltransferases and cinnamyl alcohol dehydrogenases.

Table S2. Primers used for the endophytic quantification of *V. dahliae*.

Table S3. Primers used in the ChIP assay.

Table S4. Primers used for gene expression analysis.

Fig. S1. Immunity phenotypes of P2 plants against *V. dahliae*.

Fig. S2. Verticillium wilt symptoms caused on mutants of the GNAT and MYST families.

Fig. S3. Verticillium wilt symptoms caused on mutants of the CBP family.

Fig. S4. Histone acetyltransferases contribute to the K165-mediated biocontrol activity and the establishment of inherited resistance to *V. dahliae*.

Fig. S5. Identification of pathways involved in the biocontrol activity and the establishment of inherited resistance to *V. dahliae*.

Fig. S6. K165-mediated transcription of defence and lignin biosynthesis genes in seedlings.

Fig. S7. Lignin levels and immunity phenotype of the *cad3* mutant.

erab154_suppl_Supplementary_Materials

## Data Availability

All data supporting the findings of this study are available within the paper and within its supplementary data published online.
