# Supplementary material for: The bacterial biocontrol agent Paenibacillus alvei K165 confers inherited resistance to Verticillium dahliae
Source: J Exp Bot. 2021 Apr 8;72(12):4565–76. doi: 10.1093/jxb/erab154 (PMC8163062; doi:10.1093/jxb/erab154)
Supplement: erab154_suppl_Supplementary_Materials [file erab154_suppl_supplementary_materials.pdf]

Table S1 Mutant lines of histone acetyltransferases and cinnamyl alcohol dehydrogenases

Gene	Gene locus	Mutant	T-DNA insertion lines	Stock number
HAG1	At3g54610	*gnc5-5*	SALK_048427	N548427
HAG2	At5g56740	*hag2-99*	SALK_051832C	N669699
HAG4	At5g64610	*hag4-96*	SALK_027726C	N655396
HAG5	At5g09740	*hag5-75*	SALK_106046C	N660075
HAC1	At1g79000	*hac1-06*	SALK_080380C	N671606
HAC4	At1g55970	*hac4-15*	SALK_051750C	N670715
HAC5	At3g12980	*hac5-78*	SALK_152684C	N664378
HAC12	At1g16710	*hac12-04*	SALK_071102	N25104
CAD3	At2g21890	*cad3*	SALK_007154	N507154

Table S2 Primers used for the endophytic quantification of *V. dahliae*

		V. dahliae	
ITS1/ITS2 5.8S rRNA	Z29511	5′-CCGCCGGTCCATCAGTCTCTCTGTTTATAC-3′<br>5′-CGCCTGCGGGACTCCGATGCGAGCTGTAAC-3′	Pantelides et al., 2010
		A. thaliana	
RHIP1	AT4G26410	5′-GAGCTGAAGTGGCTTCCATGAC-3′<br>5′-GGTCCGACATACCCATGATCC-3′	Czechowski et al., 2005

Table S3 Primers used in the ChIP assays

Gene	Gene locus	Primer position	Primer sequence
PR1	AT2g14610	Promoter (A)	5'-CATTTGGACAATTGCAATGAA-3'
			5'-TTTGGGGTTCGTAAACATCG-3'
		5' end of open reading frame (B)	5'-TGTTTACGAACCCCAAAATCA-3'
			5'-GAGGGAAGAACAAGAGCACCT-3'
PDF1.2	At5g44420	Promoter (A)	5'-ATGTGTGGGGTTACCACGTT-3'
			5'-CGGCTGGTTAATCTGAATGG-3'
		5' end of open reading frame (B)	5'-ACTTGTCCTAACCGCGAGAA-3'
			5'-CGGCTGGTTAATCTGAATGG-3'
NPR1	AT1g64280	Promoter(A)	5'-CTCGTTGACTTGACTTGGCTC-3'
			5'-AGAGGAGTCGGTGTTATCGG-3'
		open reading frame(B)	5'-GAGATCGCCGAAATGAAGGGA-3'
			5'-AGTCCGATGAAGTGGGTGAGA-3'
CAD3	At2g21890	Promoter(A)	5'-AGGTTCCTTTCGTTTACTTTTGAGTT-3'
			5'-GGGTAACGAGAGAATCCCCAA-3'
		open reading frame(B)	5'-CACTCGACCTGCCAATTTTCC-3'
			5'-CAGTTACTTCCACAACACGTCA-3'
CAD4	AT3g19450	Promoter(A)	5'-TACCGTCGTCCACATTGTTTT-3'
			5'-GTAAGCTCGACCAACCAAACCT-3'
		open reading frame(B)	5'-CACACGCACACACCTTGTTTC-3'
			5'-CACTCGCCATTAGACCGAAGT-3'

Table S4 Primers used for gene expression analysis

Gene	Gene locus	Primer sequence	Citation
PR1	AT2G14610	5′-TCACAACCAGGCACGAGGAG-3′<br>5′-CACCGCTACCCCAGGCTAAG-3′	Pantelides et al., 2010
PDF1.2	At5g44420	5′-CTGTTACGTCCCATGTTAAATCTACC-3′<br>5′-CAACGGGAAAATAAACATTAAAACAG-3	Pantelides et al., 2010
NPR1	AT1G64280	5'-GTCTTCTCCGCAAGCCAGTTGA-3'<br>5'-AACCGTGGAACTCGGGAAACGA-3'	Pantelides et al., 2010
CAD3	At2g21890	5'-CGACCTGCCAATTTTCTCTCTA -3'<br>5'-TCAGAATCTAGACGTGATCG-3'	Raes et al., 2003
CAD4	AT3g19450	5'-CTTCGGTCTAATGGCGAGT-3'<br>5'-CGAGAGGGGTCACGAAC-3'	Primer-BLAST
RHIP1	AT4G26410	5'-GAGCTGAAGTGGCTTCCATGAC-3'<br>5'-GGTCCGACATACCCATGATCC-3'	Czechowski et al., 2005

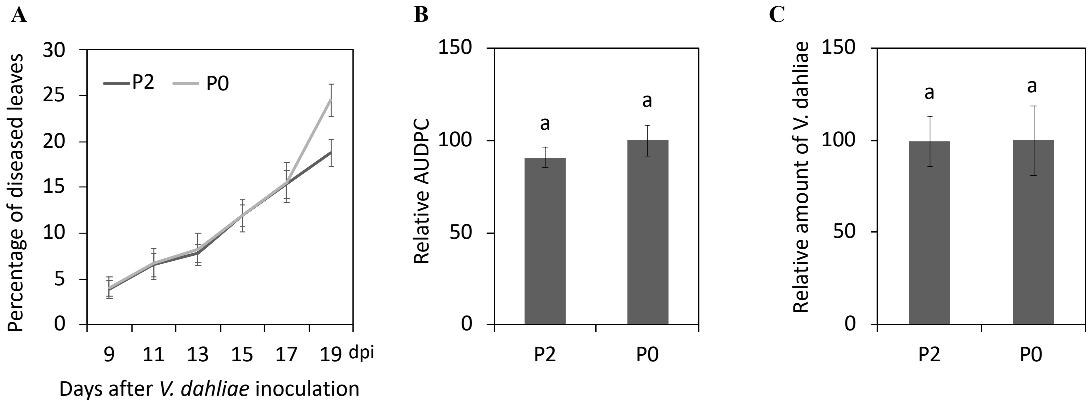

Fig. S1 Immunity phenotypes of P2 plants against *V. dahliae*. (A) The immunity phenotypes of untreated controls plants (P0) and offspring of P1 plants (P2) are expressed as percentage of diseased leaves starting at 9 days post inoculation (dpi) and were assessed every two days until 19 dpi. **(B)** The area under the disease progress curve (AUDPC) was calculated as a percentage of the disease of the untreated controls (P0) plants. The experiments were repeated three times (n=10). **(C)** The relative endophytic level of the *V. dahliae* DNA in P2 and P0 plants was determined at 19 dpi as a percentage of the fungal level in P0 plants. Five biological repeats were conducted (n=6). Error bars represent +/- one SE and different letters denote significance differences using t-test, P<0.05.

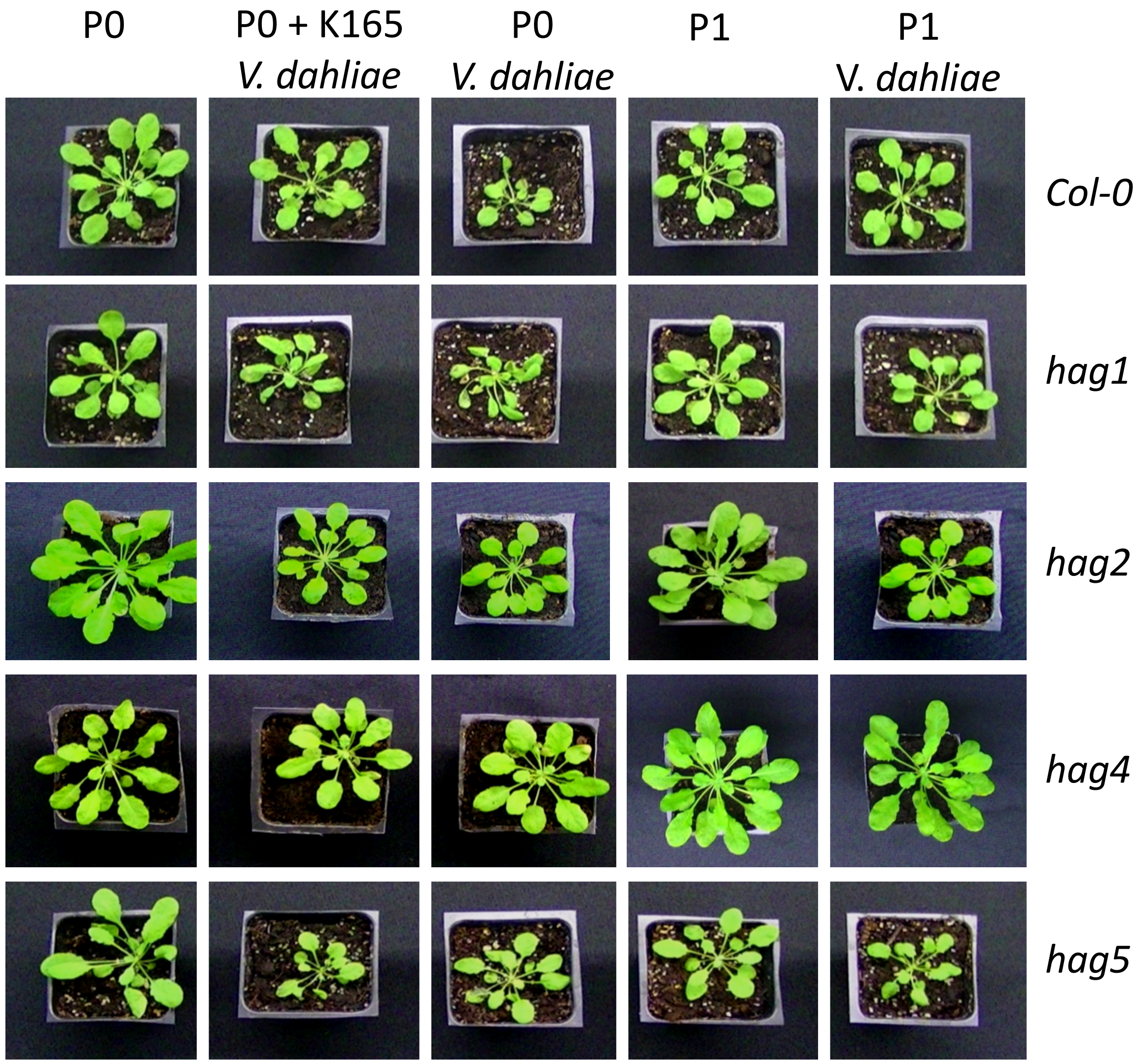

Fig. S2 Verticillium wilt symptoms caused on mutants of the GNAT and MYST families. Untreated controls plants (P0), K165-treeated P0 (P0+K165) and offspring of K165-treated P0 (P1) plants of the indicated mutants and wildtype Col-0 plants were inoculated with *V. dahliae*. The photograph was taken at 19 days post-inoculation with *V. dahliae*.

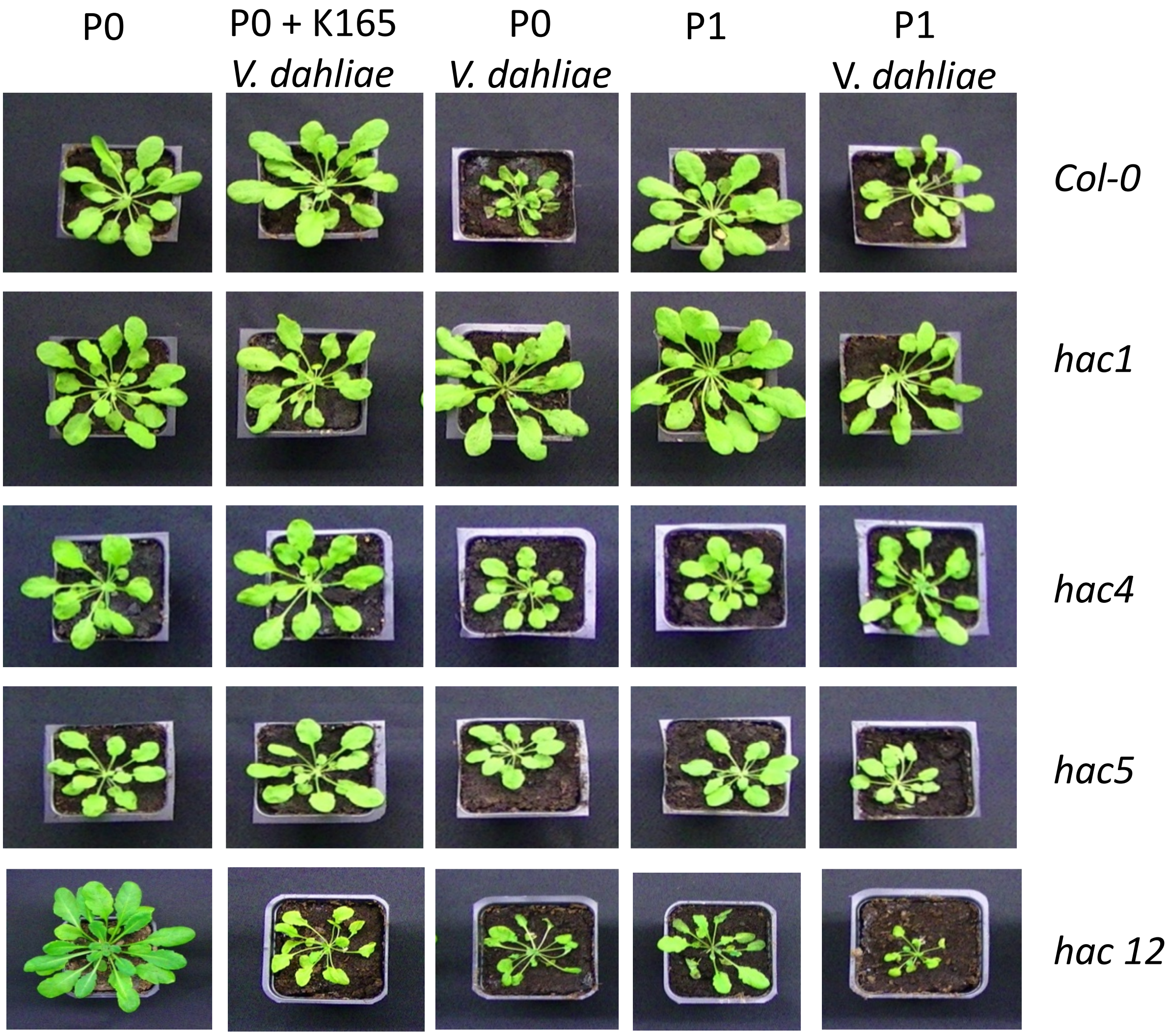

Fig. S3 Verticillium wilt symptoms caused on mutants of the CBP family. Untreated controls plants (P0), K165-treeated P0 (P0 + K165) and offspring of K165-treated P0 (P1) plants of the indicated mutants and wildtype Col-0 plants were inoculated with *V. dahliae*. The photograph was taken at 19 days post-inoculation with *V. dahliae*.

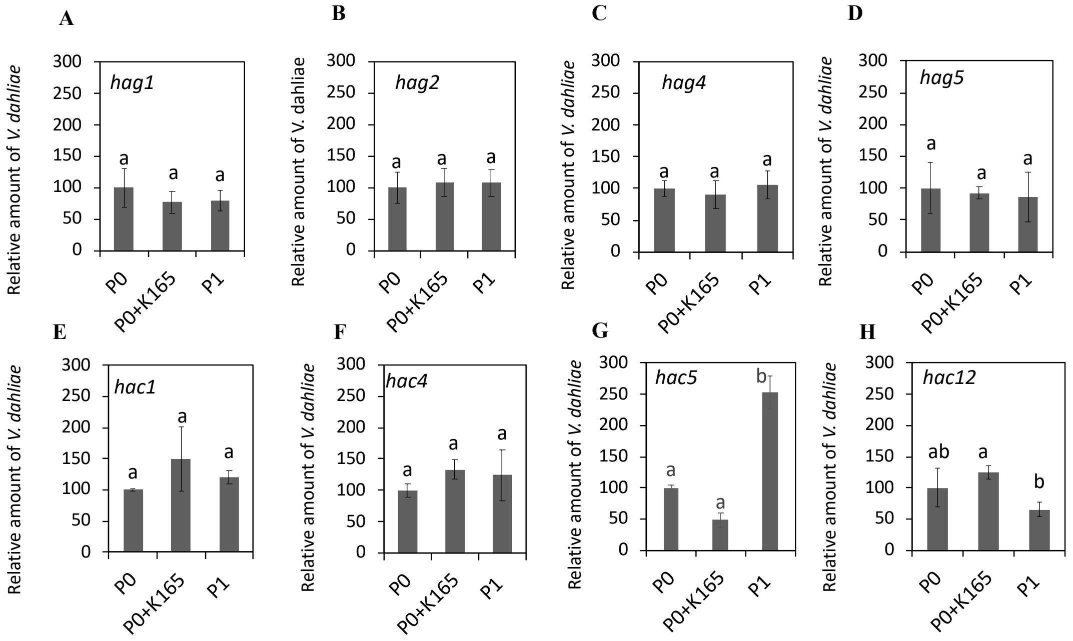

Fig. S4 Histone acetyltransferases contribute to the K165-mediated biocontrol activity and the establishment of inherited resistance against *V. dahliae.* (A-H) The relative endophytic level of the *V. dahliae* DNA in untreated controls plants (P0), K165-treated P0 (P0+K165) and offspring of K165 treated P0 (P1) plants of the of the indicated GNAT (A-B), MYST (C-D) and CBP (E-H) histone acetyltransferase mutants was determined at 28 days post inoculation as a percentage of the fungal level in P0 plants. Five biological repeats were conducted (n=6). Error bars represent +/- SE and different letters denote significance differences according to analysis of variance (ANOVA), followed by Tukey's multiple range test P<0.05.

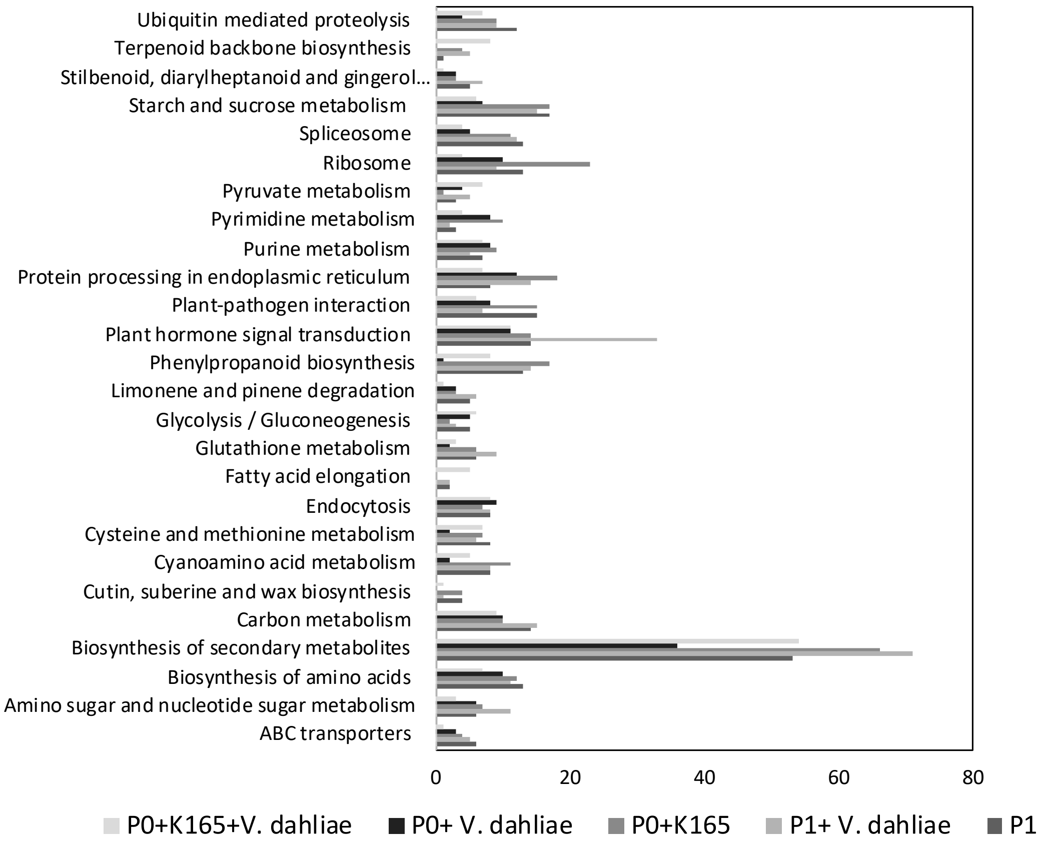

Fig. S5 Identification of pathways involved in the biocontrol activity and the establishment of inherited resistance against *V. dahliae.*

Gene expression changes of untreated controls plants (P0), K165-treated P0 plant (P0+K165) and offspring of K165-treated P0 plants (P1) at 0- and 3-days post inoculation with *V. dahliae* were quantified. Differentially expressed genes with fold change greater than 1.5-fold compared to P0 plants (ANOVA P-value < 0.05) were annotated to pathways using the KEGG mapper tool.

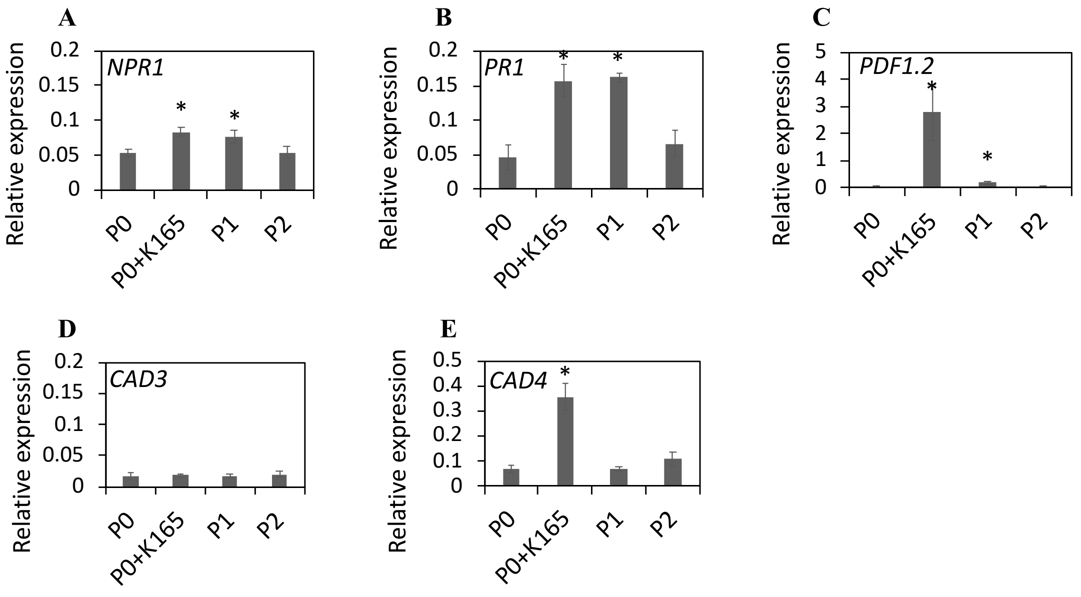

Fig. S6 K165-mediated transcription of defence and lignin biosynthesis genes in seedlings. Gene expression levels of *NPR1* **(A)**, *PR1* **(B)**, *PDF1.2* **(C)**, *CAD3* **(D)** and *CAD4* **(E)** in untreated controls plants (P0), K165-treated P0 plant (P0+K165), offspring of K165-treated P0 plants (P1) and offspring of P1 plants (P2). Relative expression levels of the indicated genes to a housekeeping gene (*AT4G26410*) are shown. Values are means +/- SE, n = 10. Experiments were performed three times with similar results and asterisks (*) denote significance differences to P0-untreated relative expression levels using t-test, $P<0.05$

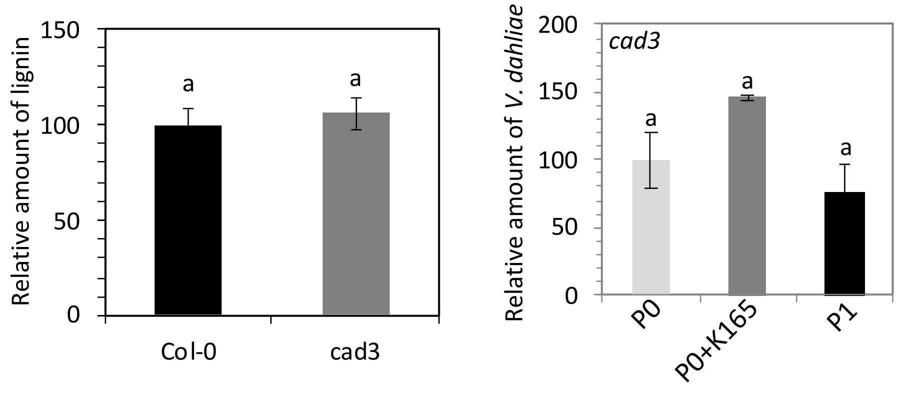

Fig. S7 Lignin levels and immunity phenotype of *cad3* mutant

(A) Relative lignin levels in untreated controls plants (P0) of Col-0 and *cad3* plants. Relative lignin levels were calculated as a percentage of the lignin levels of the Col-0 P0 plants. Different letters denote significance differences according to analysis of variance (ANOVA), followed by Tukey's multi§ple range test P<0.05. All experiments were repeated five times (n=5) with similar results and error bars represent +/- SE. **(B)** The relative endophytic level of *V. dahliae* DNA in P0, K165-treated P0 (P0+K165) and offspring of K165-treated P0 (P1) *cad3* plants were determined at 19 days post inoculation as a percentage of the fungal level in P0 plants. The experiment was repeated three times (n=6). Error bars represent +/- SE and different letters denote significance differences using t-test, P<0.05.
